# Analysis of expressed SNPs identifies variable extents of expression from the human inactive X chromosome

**DOI:** 10.1186/gb-2013-14-11-r122

**Published:** 2013-11-01

**Authors:** Allison M Cotton, Bing Ge, Nicholas Light, Veronique Adoue, Tomi Pastinen, Carolyn J Brown

**Affiliations:** 1 Department of Medical Genetics, Molecular Epigenetics Group, Life Sciences Institute, University of British Columbia, Vancouver, BC V6T 1Z3, Canada; 2 McGill University and Genome Québec Innovation Centre, Department of Human and Medical Genetics, McGill University, Montréal, Québec H3A 0G1, Canada

## Abstract

**Background:**

X-chromosome inactivation (XCI) results in the silencing of most genes on one X chromosome, yielding mono-allelic expression in individual cells. However, random XCI results in expression of both alleles in most females. Allelic imbalances have been used genome-wide to detect mono-allelically expressed genes. Analysis of X-linked allelic imbalance in females with skewed XCI offers the opportunity to identify genes that escape XCI with bi-allelic expression in contrast to those with mono-allelic expression and which are therefore subject to XCI.

**Results:**

We determine XCI status for 409 genes, all of which have at least five informative females in our dataset. The majority of genes are subject to XCI and genes that escape from XCI show a continuum of expression from the inactive X. Inactive X expression corresponds to differences in the level of histone modification detected by allelic imbalance after chromatin immunoprecipitation. Differences in XCI between populations and between cell lines derived from different tissues are observed.

**Conclusions:**

We demonstrate that allelic imbalance can be used to determine an inactivation status for X-linked genes, even without completely non-random XCI. There is a range of expression from the inactive X. Genes escaping XCI, including those that do so in only a subset of females, cluster together, demonstrating that XCI and location on the X chromosome are related. In addition to revealing mechanisms involved in *cis*-gene regulation, determining which genes escape XCI can expand our understanding of the contributions of X-linked genes to sexual dimorphism.

## Background

Regulatory elements controlling gene expression can lie long distances from the transcription start site (TSS), further complicating the already challenging task of identifying comparatively small sequence elements that modulate expression patterns. An important new global approach to the understanding of gene regulation by *cis*-acting regulatory elements is the determination of allelic imbalances (AIs) between two polymorphisms on homologous chromosomes through genome-wide methodologies. Both cDNA microarrays that detect single nucleotide polymorphisms (SNPs) and RNA sequencing have shown that a surprising 10% or more of loci show AI, implicating differences in regulatory sequences between the two alleles [1,2]. Heritable variation in expression is believed to bring about many disease predispositions, generating substantial interest in identifying sequences underlying such variation (reviewed in [3]). One of the most dramatic examples of long-range silencing is X-chromosome inactivation (XCI), which occurs early in mammalian development to equalize expression of X-linked genes between the two X chromosomes of females and the single X chromosome of males. The majority of autosomal genes are believed to be bi-allelically expressed, whereas X-linked genes are generally mono-allelically expressed within a single cell. In females with random XCI, expression is observed from both the paternal and maternal X chromosome due to expression of each allele in different cell populations. Overall this results in a bi-allelic expression pattern for the majority of X-linked genes. If cells with either the maternal or paternal X chromosome inactivated are more frequent in the population assayed, a high AI for X-linked genes subject to XCI will result. As XCI is stably inherited through mitosis, skewing of XCI can occur by chance when a limited number of precursor cells give rise to a population or due to selective proliferation of cells with one or the other X active (reviewed in [4]). Previous studies of AI have observed an elevated frequency of AI on the X chromosome, which was attributed to the partial clonality of the cells being assessed, particularly for lymphoblastoid cell lines (LCLs) [1,5]. Therefore, the X chromosome is often excluded from AI analysis; however, AI of X-linked genes can inform our understanding of XCI, which in turn contributes to understanding long-range *cis*-regulatory processes.

XCI is a remarkable example of epigenetic silencing, in which an approximately 160 Mb chromosome containing almost 1,000 genes is silenced to become the inactive X chromosome (Xi). Inactivation spreads in *cis* from a single X inactivation center, such that only one of the two essentially identical X chromosomes is silenced in any given normal female cell. It is known that the expression of XIST, a long non-coding RNA, is essential for the initiation and spread of silencing, likely through the recruitment of multiple chromatin remodeling complexes (reviewed in [6]) and the engagement of *cis*-acting DNA receptor sequences [7,8]. The Xi and the active X chromosome (Xa) differ with respect to the overall enrichment of histone modifications. As would be expected given the highly heterochromatic nature of the silent Xi, it is generally enriched for inactive histone modifications and depleted for active histone modifications (reviewed in [6]). These epigenetic marks contribute co-operatively to the remarkably stable inheritance of the silenced state over subsequent somatic cell divisions. Surprisingly, however, not all genes on the Xi are silenced as approximately 15% of X-linked genes have been reported to continue to be expressed from both the Xa and the Xi. Identification of such 'escapees’ has been made predominantly through the use of somatic cell hybrids in which the human Xa and Xi can be isolated apart from each other in a mouse background, thereby allowing direct assessment of expression from the Xi. The list of genes that escape from XCI assessed in this way has been confirmed or extended by the analysis of expressed polymorphisms. Except in the rare circumstance where presence of a heterodimer indicates bi-allelic expression (for example, *G6PD*[9]), allelic expression needs to be examined either at the single cell level, or in clonal populations of cells where the same X chromosome is always the Xa, in order to determine if there is expression from the Xi. A threshold of 10% expression from the Xi relative to that observed from the Xa has often been used to define a gene as one that escapes from XCI [10,11]. In addition, escapees have been shown to lack the heterochromatic marks found on inactivated genes. These marks, in particular DNA methylation, have been used as a surrogate to determine whether a gene is subject to XCI. Studies of the XCI status of genes in multiple tissues have been limited, but evidence is accumulating for the presence of variability between tissues for individual genes [12] and more broadly using DNA methylation as a mark of inactivation status [13,14], or allelic gene expression in mouse models [11]. In addition, it has been shown that some genes escape XCI in some females, but are subject to XCI in other females (for example, *CHM*[15], *TIMP1*[16]), a finding that is also extended by DNA methylation-based studies [13] as well as chromatin immunoprecipitation (ChIP)-sequencing for RNA polymerase [17].

The mechanism by which genes escape from XCI remains to be determined; however, there is evidence to suggest that some genes may escape due to the presence of an intrinsic DNA escape element [18]. Furthermore, domains of subject and escape genes are proposed to be segregated by boundary elements including CTCF [19]. Generation of a more complete catalog of inactivation status for X-linked genes may provide insights into the nature of such elements, and whether they differ between females or tissues. Here we seek to use X-linked AI data to expand the list of X-linked genes with known XCI statuses and to better assess the level of Xi expression in an effort to further our understanding of how *cis*-acting silencing occurs.

## Results and discussion

### Training sets demonstrate that AI reflects XCI status in females

**Figure 1:**
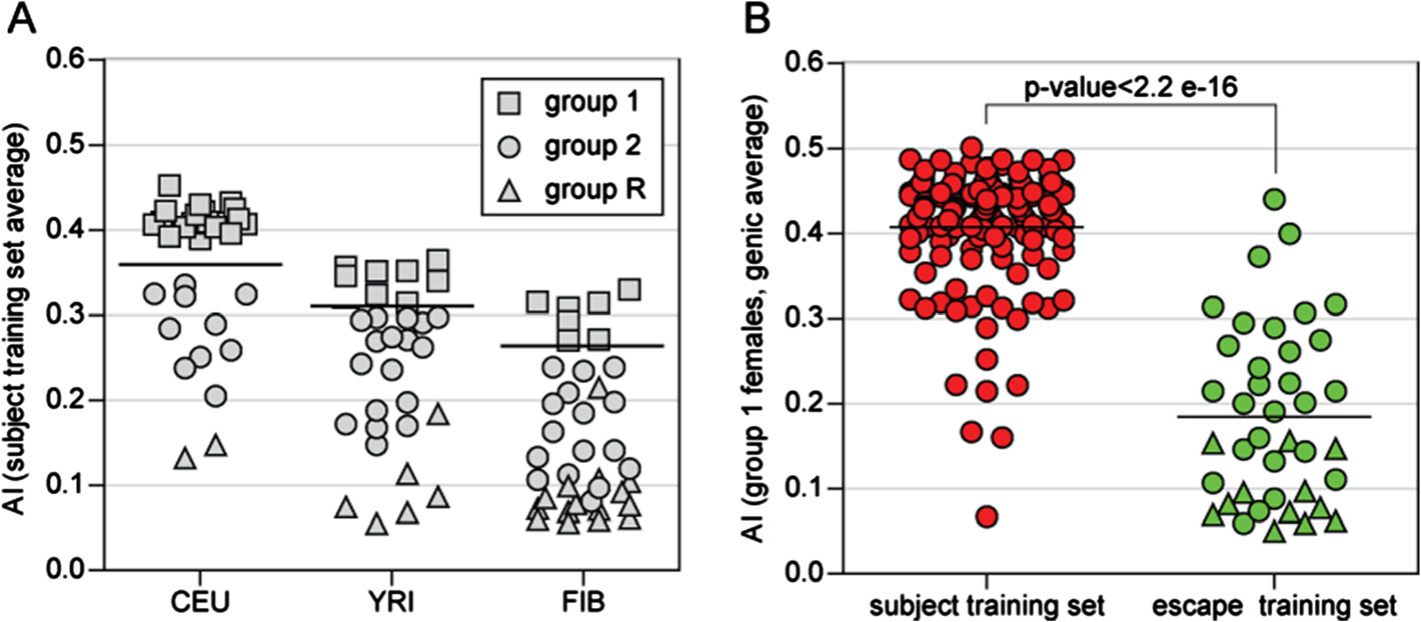
**Genes that escape XCI have significantly lower AIs than genes that are subject to XCI. (A)** The degree of skewing of XCI varies between sample sets. To determine the degree of skewing all genes from the subject training set were examined (minimum of two probes per gene) and the average AI per gene determined (only probes with a total cDNA greater than the sample set threshold; Additional file 2). The thick black line shows the AI at which 99.5% of autosomal genes are found. Females with an average AI from the subject training set above this threshold were classified as the most highly skewed females in each population (group 1, squares), females below this threshold were classified as either group 2 (circles) or group R (triangles). **(B)** Examining group 1 females only, genes from the escape training set (green) have a significantly (*P*-value <2.2 e-16) lower genic AI than genes from the subject training set (red). Genes located in the Xp pseudoautosomal region (PAR1) are shown as triangles while circles represent non-PAR1 genes.

In order to determine if AI could be utilized to identify genes that escape from XCI, we analyzed previously generated AI data from three sample sets, 54 (male n = 24, female n = 30) LCLs from the Centre d’Etude du Polymorphisme Humain (CEPH) HapMap population (herein referred to as CEU), 61 (male n = 30, female n = 31) LCLs from the Yoruban HapMap population (herein referred to as YRI), and 75 (male n = 37, female n = 38) fibroblast cell lines (herein referred to as FIBs) [1,2] (J. Wagner *et al.*, manuscript in preparation). We anticipated that there would be appreciably higher AI for genes subject to XCI only if the female analyzed showed substantial skewing of XCI. To identify such females we derived a set of genes that were previously reported to be subject to XCI by both expression analysis and DNA methylation analysis in multiple tissues [10,13]. This yielded a set of 177 genes (Additional file 1) referred to as the subject training set. Averaging of the AI calculated for genes from this training set with two or more informative probes showed a range of average AI values from 0.0670 to 0.4751, where AI values represent a fractional deviation from 50:50 allele ratio (0 equals perfect bi-allelic expression and 0.5 indicates mono-allelic expression in a female with completely skewed XCI). Over half (18 of the 30) of the CEU LCLs, 8 of the 31 YRI LCLs and 7 of the 38 FIB samples showed an average AI for these subject genes above the threshold at which only 0.5% of the autosomal probes for that sample set were observed. We classified those females for which the average AI fell above the threshold as group 1 females (squares in Figure 1A), those females with less, but still significant evidence for skewing of inactivation as group 2 females and those females with essentially random inactivation as group R females (see Materials and methods; Additional file 2). Gimelbrant *et al.*[20] previously detected substantial skewing in CEU samples consistent with our results showing considerable skewing of XCI in LCLs and the CEU samples showing more skewing than the YRI samples.

By examining only the group 1 females, we tested our assumption that AI would reflect the XCI status of genes by establishing a second training set consisting of genes that escaped from XCI. These included 15 genes from the Xp pseudoautosomal region (PAR1) and 28 non-PAR1 genes (Additional file 3) for which there was concordance between the expression data [10] and DNA methylation in multiple tissues [13]. Given the small number of group 1 females for each population and the fact that only a limited number of females were informative at any gene, we wished to maximize the sample size by analyzing all three sample sets together. Overall, the average AI from the combined group 1 females for the escape training set was 0.1845, while the average AI for the subject training set was significantly (*P*-value < 2.2 e^-16^) higher at 0.4112, supporting the use of AI to identify genes that escape from XCI. The subject and escape training sets were significantly different regardless of which combination of females was used (group 2 females only *P*-value = 3.778 e^-16^, group 1 and 2 females *P*-value = 8.273 e^-16^), demonstrating that AI can be used to distinguish genes that escape from XCI from genes that are subject to XCI. Interestingly, however, there was overlap between the two distributions (Figure 1B), and we explore the source of such heterogeneity in a later section. Having established that AI could distinguish genes that are subject to XCI from those that escape from XCI we set out to predict an XCI status for genes across the X chromosome.

It should be noted that there are some limitations to using expressed SNPs to determine XCI status. First, only probes for which there are informative SNPs in a given female could be assessed. For the escape and subject training sets, respectively, 75% (1,093/1,457) to 88% (3,006/3,415) of probes were informative in at least one female. Second, as with any analysis of cDNA, genic expression levels vary greatly between genes and some genes may not be expressed at a high enough level for reliable detection of AI. SNPs that are homozygous and therefore uninformative are expected to have an AI of zero since there cannot be a bias in expression from two alleles if only one allele is present. However, for poorly expressed genes with a low cDNA signal intensity, the AI could be above zero due to inconsistent hybridization. The minimum total cDNA threshold (vertical line in Additional file 4) was established by statistically determining the total cDNA level at which most probes from uninformative females showed an unexpected (greater than 0) AI. Incorporating the minimum total cDNA thresholds reduced the number of analyzed informative probes to 68% (2,331/3,415) in the subject training set and 52% (754/1,457) in the escape training set. Third, the calculation of allelic expression can be impacted by skewed hybridization intensities in genomic DNA of heterozygotes. Therefore, we discarded those probes that consistently showed a genomic DNA ratio of greater than 0.7 (see further details in Materials and methods). In order for a gene to be examined we required that a female have at least two informative probes. We were interested in the frequency at which multiple probes within the same gene showed different levels of AI as possible evidence for biologically variable processes, such as alternative spliced transcripts demonstrating different XCI statuses. Genes with only two probes had, on average, four informative females and across all females, in 83% of these genes the two probes showed concordant AIs. A Chi-square analysis of all genes with two probes and four informative females found no genes that were significantly enriched for discordant probes (*P*-values 0.8164 to 0.9112), suggesting experimental noise rather than biological sources, such as alternative splicing, as the cause of discordant probes. After taking into account the requirement that a probe be above the total cDNA threshold, that at least two probes be present in a gene and that none of these probes were above 0.7 in the genomic DNA, 79% (140/177) of genes in the subject and 93% (40/43) of genes in the escape training set were able to be examined, demonstrating that while our criteria for analysis are stringent, AI differences between the subject and escape training sets can still be detected for a substantial proportion of genes, and thus AI could be used to characterize XCI status across the X chromosome.

### The majority of X-linked genes examined are subject to XCI

**Figure 2:**
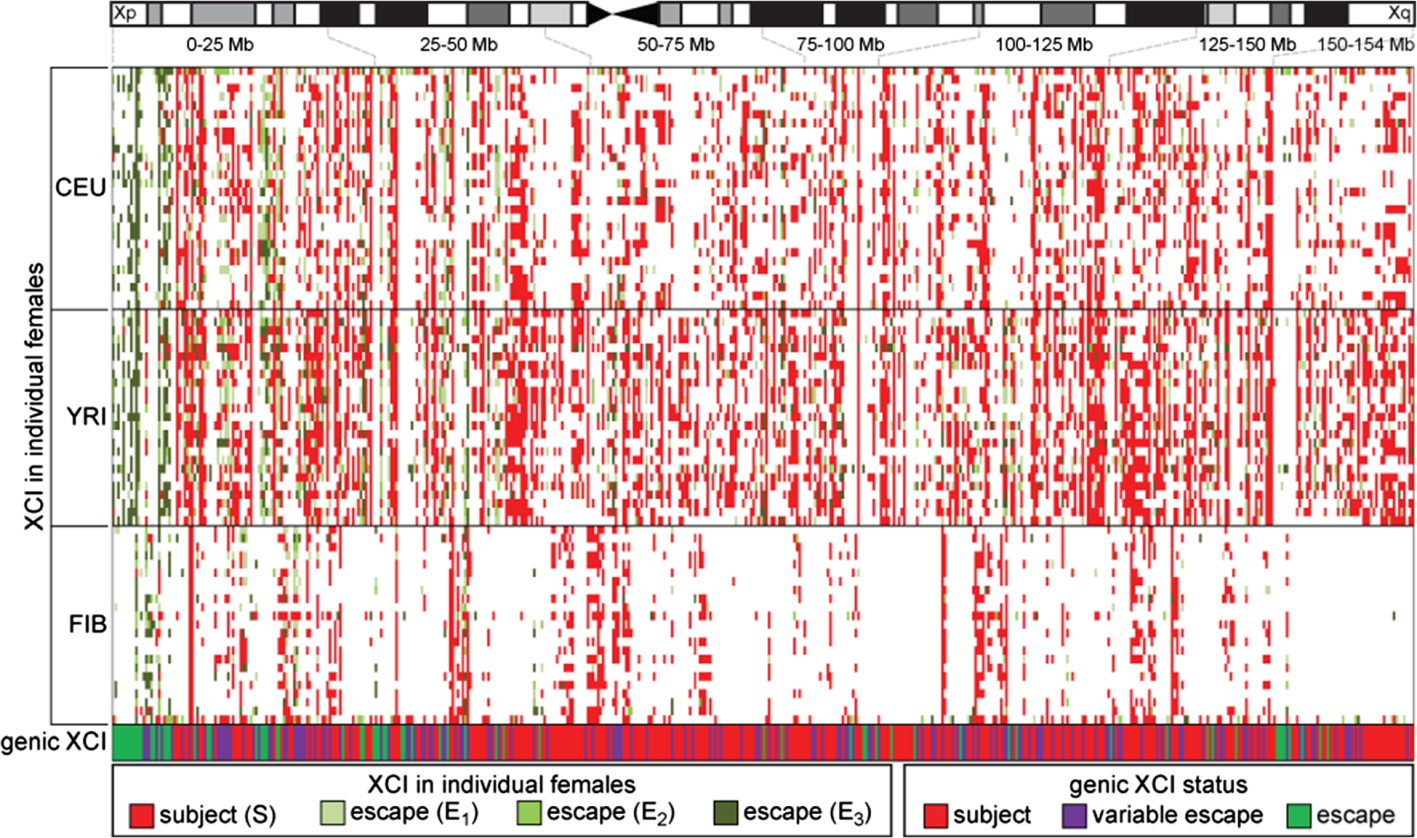
**The majority of genes are subject to XCI.** XCI status in individual informative females with each column representing one gene (only genes with at least one informative female are included) and each row a single female (group 1 and group 2 females only) from the three sample sets. The XCI status of each female is either subject to XCI (red) or escapes from XCI (E_3_, light green; E_2_, bright green; E_1_, dark green). For how AIs were converted into XCI status, see Additional files 2, 5, 9 and 12. The genic XCI status was determined by calculating the percentage of females that escaped from XCI for each gene. Subject to XCI (red: 0 to 22% of informative females escaped from XCI), variable escape from XCI (purple: 22 to 78% of informative females escaped from XCI) and escape from XCI (green: 78 to 100% of informative females escaped from XCI). An ideogram of the X chromosome is shown with 25 Mb regions shown with grey dotted lines.

In order to maximize the ability to determine an XCI status for as many genes as possible, we extended our analysis to include the group 2 females with partially skewed XCI. Therefore, we needed to adjust for the estimated degree of skewing of XCI in the females, as determined with the subject training set (see Materials and methods; Additional files 2 and 5). Using these adjusted thresholds, each gene with two or more informative SNPs in a female was assigned an XCI status. Subject genes were classified as those genes showing less than 10% Xi expression relative to the Xa expression level. Given the range of AIs noted in the escape training set in Figure 1, escape from XCI was subdivided into three levels (E_1_, E_2_ and E_3_), with E_1_ having the highest expression from the Xi. This analysis clearly demonstrates that the majority of genes are subject to XCI (Figure 2). Not unexpectedly, the YRI have more informative genes than either the CEU or FIB sample sets [21]. The lower informativity in the FIB samples is attributable to a greater elimination of probes for analysis using our cDNA thresholds (Additional file 6).

The largest study of inactivation status to date is that of Carrel and Willard [10], in which expression in 7/9 Xi somatic cell hybrids (or 78% of hybrids) was used to classify a gene as escaping from XCI. Thus, to combine the XCI status from individual females into a genic XCI, we have used the threshold that if more than 78% of the informative females show an AI consistent with at least 10% expression from the Xi, then the gene is called escape. Using these definitions 58% (n = 294) of all genes examined were subject to XCI (less than 22% of females escaped from XCI), 13% (n = 68) of genes escape from XCI, while 29% (n = 148) of genes were found to show variable escape from XCI (22% to 78% of individual females escape from XCI) (Figure 2). We were able to assign an XCI status to 115 genes for which one was not previously determined. Of these, 46 were subject to XCI, 29 escaped from XCI while 40 showed variable escape from XCI.

While an AI score is a useful measure of the imbalance between the expression levels of the two alleles, it is not an intuitive value and does not take into account the level of skewing in each female. Therefore, we converted all AIs in group 1 and 2 females to the percentage of Xi expression as a ratio of Xa expression (hereafter referred to as %Xi). To convert an AI score into a %Xi value the level of skewing of XCI was used for each female. Skewing has traditionally been determined using the androgen receptor assay [22], which shows good correlation with expression-based determination of skewing [23]. A lack of agreement between some assays highlights the perils of using only a single gene to determine skewing [24]. To address this we instead averaged up to 177 genes (the subject training set) to determine the degree of skewing. The use of a subject training set rather than individual genes reduces noise in AI. While genes that are subject to XCI are expressed only from the Xa, *XIST* shows mono-allelic expression, but is expressed from the only Xi and can be used to estimate skewing. Only 12 females were informative for at least 2 SNPs within *XIST*; therefore, the subject training set allowed for the degree of skewing to be determined in a greater proportion of females. The degree of skewing predicted by XIST was highly correlated (data not shown, R[2] = 0.8323, *P*-value <0.0001) with the degree of skewing determined by the subject training set. Any method to determine the degree of skewing of XCI is reliant on the assumption that either a gene that is subject to XCI is completely silenced on the Xi or is completely methylated on the Xi. Just as the androgen receptor assay is less reliable at determining skewing when DNA methylation of the Xi is incomplete [24], our means of determining skewing will underestimate the degree of skewing if there is any Xi expression from our subject training set genes. This would in turn cause the conversion of AI scores into XCI statuses to be slightly off. As a result, genes that had an AI greater than the average AI from the subject training set translate into a negative %Xi expression level. These negative values were treated as 0% Xi expression as they are likely the result of an underestimation of skewing. Regardless of how skewing of XCI is ultimately determined, the inclusion of females with slightly skewed XCI allows for more females to be examined, therefore resulting in a more complete atlas of expression levels from the Xi.

### Xi demonstrates a continuum of expression levels

**Figure 3:**
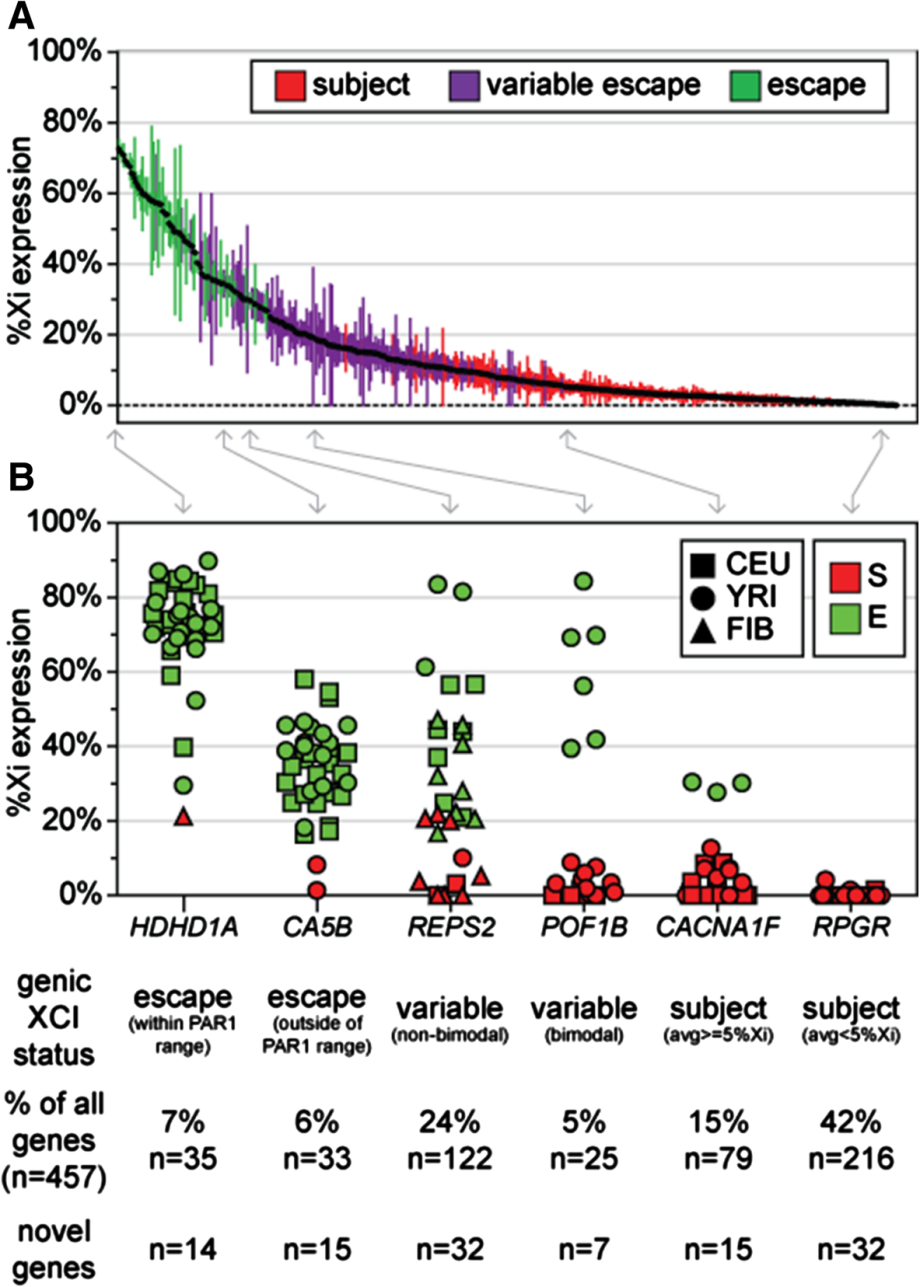
**Distribution of average %Xi expression levels shows a range of expression. (A)** Ranked average %Xi expression (highest to left, lowest to right). Error bars represent the standard error of the mean while the color indicates the assigned genic XCI status. **(B)** For each gene, informative females are represented with a different shape based on the sample set (CEU, square; YRI, circle; FIB, triangle) and color based on XCI status (E, green; S, red). Note that the E and S ranges overlap because the E:S boundary differs in each female based on skewing. The genic XCI status was determined by calculating the percentage of females that escaped from XCI for each gene: subject (red), 0 to 22% of informative females escaped from XCI; variable escape (purple), 22 to 78% of informative females escaped from XCI: escape (green), 78 to 100% of informative females escaped from XCI. The number and percentage of all genes is given below each example as well as the number of novel calls in each category. The number of informative females for each example gene is: *HDHD1A* n = 39, *CA5B* n = 36, *REPS2* n = 30, *POF1B* n = 22, *CACNA1F* n = 32 and *RPGR* n = 41.

Genes were ranked from those with the highest %Xi expression (escape genes) to those with the lowest (subject genes) and graphed along with the standard error of the mean between females (Figure 3A). The largest cluster of genes that escape from XCI was found in the PAR1 and these genes are anticipated to have full expression from the Xi, as they are identical between the X and Y chromosomes. In this study, the informative PAR1 genes (n = 11) showed an average %Xi expression from 72.63% (*P2RY8* with 33 informative females) to 49.16% (*PLCXD1* with 16 informative females). The %Xi expression of the PAR1 genes was not 100%, suggesting that not even PAR1 genes show Xi expression equivalent to the Xa expression level, although complete dosage compensation of these genes may still be achieved through modulating the expression of the Y chromosome copy [25]. Although PAR1 genes had a greater %Xi expression than non-PAR1 genes, 24 non-PAR1 genes had an average %Xi expression within the PAR1 range (*HDHD1A* given as an example in Figure 3B). These 24 genes are therefore the best examples of genes that show a consistently high degree of Xi expression without being located in the PAR1. Nine of the 24 non-PAR1 genes were not previously examined by expression analysis or DNA methylation and therefore are novel genes that escape from XCI [10,13]. In 3 of the 24 non-PAR1 genes (*LANCL3*, *CXorf41* and *GABRE*), both previous expression and DNA methylation evidence would suggest that these genes are actually subject to XCI. *GABRE*, was actually a member of the subject training set. Although *GABRE* had a high average %Xi expression level, it was only informative in 4 females in our study and had a low average total cDNA (CEU, 1,521; YRI, 5,231), just above the minimum total cDNA threshold (CEU, 1,020; YRI, 4,174). It is therefore likely *GABRE* is in fact not expressed at high enough level in enough informative females to accurately determine the XCI status. It should be cautioned that of the 24 non-PAR1 genes with %Xi expression within the PAR1 range, 12 had fewer than 5 informative females (Additional file 7). With only a few informative females, AI due to allelic transcription differences as has been seen on the autosomes might influence our prediction of XCI. The 24 non-PAR1 genes that show a high level of expression from the Xi relative to the Xa within the PAR1 range are excellent examples of the high degree of expression possible from the Xi and should be considered when looking for model genes that escape from XCI.

Rather than grouping into clearly distinct groups of genes that escape (>10% Xi expression) or are subject (<10% Xi expression) to XCI, unexpectedly we observed a continuous distribution of expression from the Xi with an equal number of genes in each escape class (escape within the PAR1 range, n = 35; escape outside the PAR1 range, n = 33; Figure 3B). In the escape within the PAR1 range category (average genic %Xi expression = 60.63%) outliers where the gene was called subject in individual females were rare. On average, 97% of informative females were predicted to escape from XCI for each gene. In the escape outside the PAR1 range category (average genic %Xi expression = 37.31%), it was still rare that the gene was called subject in any informative females; on average, 94% of females were predicted to escape from XCI for each gene. The DNA sequences of all 57 non-PAR1 genes that were predicted to escape from XCI were put through BLAST in order to determine which genes had homology to the Y chromosome or the autosomes [26]. Over one-third (21/57 = 37%) of non-PAR1 genes predicted to escape from XCI have homology to the Y chromosome and/or the autosomes. The majority (14/21 = 67%) of genes with homology to the Y chromosome and/or the autosomes showed expression from the Xi within the PAR1 range while genes with expression outside of the PAR1 range tended (26/33 = 79%) to lack Y chromosome and/or autosome homology. Those non-PAR1 escape genes that mapped only to the X chromosome (n = 36) had a significantly (*P*-value = 0.0033) lower average genic %Xi expression level (42.70% Xi) compared to those that mapped to the Y chromosome and/or the autosomes (n = 21, 52.90% Xi). Although genes in the PAR1 escape from XCI, previous evidence would suggest that some genes (*SPRY3* and *SYBL1/VAMP7*) in the PAR2 are silenced on the Y chromosome in males and on the Xi in females while the PAR2 gene *IL9R* escapes from XCI [27]. *SPRY3* had an average genic %Xi expression of 19.38% and was predicted to show variable escape from XCI; however, it was only informative in three females. *SYBL1/VAMP7* was found to be subject to XCI in all 35 informative females with an average of 0.61% Xi. Surprisingly, *IL9R* was classified as being subject to XCI; however, 4/21 females showed some degree of escape from XCI with an average of 9.34% Xi expression.

Given the range of expression detected from the Xi at genes that escape from XCI, it is not surprising that variable escape genes also show a range of average %Xi expression levels. Genes classified as variable escape (n = 147) had an average genic %Xi expression of 18.79%, which is between that of the subject and escape genes. An intermediate average, however, could result from three quite different scenarios. First, as previously reported [15] for some genes (for example, *GYG2*), a gene may show extreme levels of Xi expression in different females and therefore be subject to XCI in some females but strongly escape from XCI in other females; we term this 'bimodal variable escape’. Second, a gene may show 'borderline variable escape’, wherein the small amount of expression from the Xi falls close to the 10% cutoff. For example, while there may not be much variation between the relative expression level from the Xi, females with 9% expression would have the gene called as subject, while those having 10% expression would have resulted in a call of escape. The third possibility is that a gene may show a broad range of Xi expression resulting in not only females that are subject to XCI as well as females that escape, but also females that escape to varying degrees. We term this 'heterogeneous variable escape’. In order to distinguish between these possibilities, we divided variable escape genes into those that had a bimodal distribution of AIs (at least 75% of informative females being either E_1_ or S, but not all E_1_ or S) and those that did not. Only 17% (n = 25) of the variable escape genes had a bimodal distribution (*POF1B* shown as an example in Figure 3B), suggesting that this was not the most common pattern of variable escape. Rather, 83% (n = 122) of variable escape genes showed a continuum of expression from the Xi (*REPS2* shown as an example in Figure 3B). For only 22% (n = 33) of variable escape genes were all of the informative females in the S or E_3_ category suggestive of the borderline variable escape category. Overall, the majority (61%, n = 89) of variable escape genes showed a pattern of XCI consistent with heterogeneous variable escape. Borderline variable escape genes had the highest average genic %Xi expression (13.07%) but the lowest average number of informative females (n = 22). Heterogeneous variable escape genes and bimodal variable escape genes had similar average genic %Xi expression (19.63% and 23.38%, respectively) and average number of informative females (n = 25). The distinction between different types of variable escape genes may provide insight into how and why escape from XCI occurs in some females but not others. Specifically, when a group of females contains different populations and/or cell lines derived from different tissues, variable escape from XCI may be suggestive of differences not based on individual females but on the features of where samples were obtained. The potential effect of sample origin will be investigated in a later section.

Genes subject to XCI (n = 295) were the largest category of genes in this study (average genic %Xi expression = 5.32%), and only rarely did subject genes include females with a call of escape from XCI with an average of 7% of informative females classified as escaping from XCI. As previously stated, traditionally a gene is classified as subject to XCI if there is less than 10% Xi expression. The majority (n = 216) of genes predicted to be subject to XCI had an average genic %Xi expression of less than 5% (*RPGR* as an example in Figure 3B) while only 79 had an average genic %Xi expression greater than 5% Xi expression (*CACNA1F* as an example in Figure 3B). Within the category of genes subject to XCI were 47 genes that had not previously been examined by either DNA methylation or expression analysis [10,13]. The large range of %Xi expression detected across X-linked genes led us to investigate how histone modifications might vary between genes with different XCI statuses.

### Extent of allelic imbalance in histone modifications reflects Xi expression level

**Figure 4:**
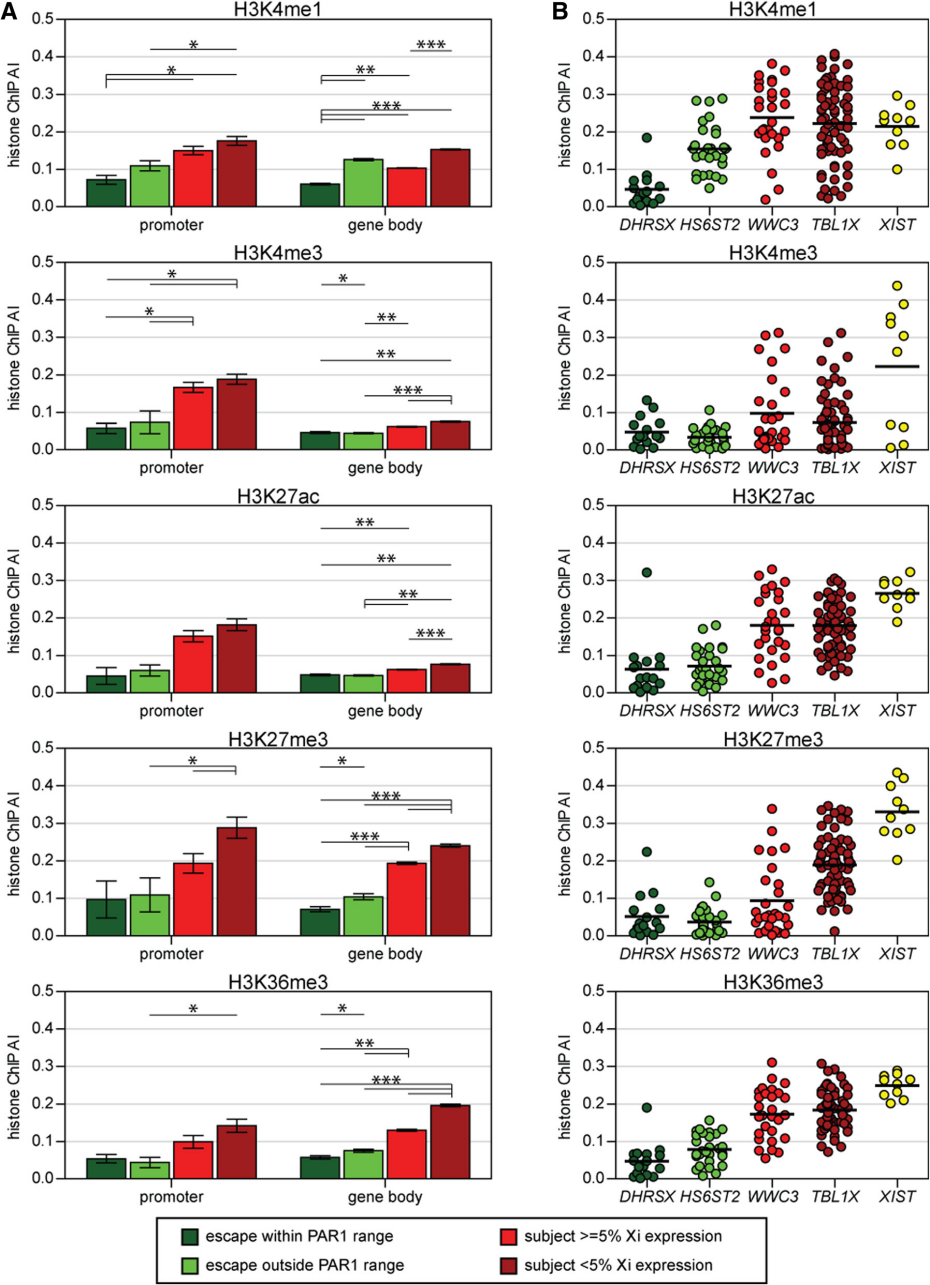
**Histone ChIP AI of individual histone modifications is generally highest at genes subject to XCI. (A)** Each histone modification is shown in a separate panel (H3K4me1, H3K4me3, H3K27ac, H3K27me3 and H3K36me3). Error bars represent the standard error of the mean while the color indicates the assigned genic XCI status: genes that escape XCI and have a %Xi expression within the PAR1 range (dark green), genes that escape XCI and have a %Xi expression outside the PAR1 range (green), genes that are subject to XCI and have a %Xi expression greater than 5%Xi (red) and genes that are subject to XCI and have a %Xi expression less than 5%Xi (dark red). **(B)** Examples of gene body histone ChIP AI of individual gene loci from each %Xi expression level along with XIST. Significant differences between means are shown as asterisks (*P-value 0.05 to 1.0 e-5, **P-value 1.0 e-5 to 1.0 e-15, ***P-value <1.0 e-15). All *P*-values were corrected for multiple comparisons.

Genes subject to XCI show an enrichment of heterochromatic modifications and a depletion of active modifications on the Xi. To determine if the intermediate levels of Xi expression also correlate with histone modifications, we created four categories of expression levels: escape genes within the PAR1 range (n = 35), escape genes outside of the PAR1 range (n = 33), subject genes with ≥5% Xi expression (n = 79) and subject genes with <5% Xi expression (n = 216). For females with non-random XCI (group 1 and 2 females) marks that differ between the Xa and the Xi should show an AI enrichment after ChIP (histone ChIP AI). We determined the average histone ChIP AI for five different histone modifications (H3K4me1, H3K4me3, H3K27ac, H3K27me3 and H3K36me3) in five different female LCL samples with skewed XCI. Two genomic regions, the promoter region (±1 kb surrounding the TSS) and the length of the gene from TSS to transcription end site were examined for each histone modification (Figure 4A). On average, the gene body contained more than 40 times as many informative probes as the promoter region; therefore, only individual gene body examples are shown in Figure 4B. These genes were selected as they contain a large number of informative probes and clearly demonstrate the differences in histone ChIP AI between the different XCI statuses. We show a combination of all histone marks in Additional file 8. At both the promoter and the gene body, genes that showed the highest level of %Xi expression (escape within the PAR1 range) showed the lowest level of histone ChIP AI while genes that showed the lowest level of %Xi expression (subject <5% Xi expression) showed the highest level of histone ChIP AI. At the promoter, the level of histone ChIP AI did not differ significantly (*P*-value = 0.2106) between the two categories of escape genes but did differ significantly (*P*-value = 0.0051) between the two categories of subject genes. The level of histone ChIP AI was significantly different between all categories in the gene body. Thus, the continuum of expression that we observe from the Xi is also observed in the extent of allele imbalance of chromatin marks in both the promoter and gene body of X-linked genes. Histone modifications play an important role in establishing large domains of silencing associated with the Xi (reviewed in [6]) and given that genes in close proximity on the linear chromosome tend to occupy the same domains, and the differences found between genes of differing %Xi expression levels, we examined the role that physical location on the X chromosome may play in influencing XCI.

### Clustering of subject and escape genes across the X chromosome

Previous reports [10,28] have found that genes that escape from XCI cluster together, particularly on the short arm of the X chromosome. To assess whether neighboring genes shared an XCI status, we first excluded the PAR1 that is known to escape from XCI and the inclusion of which would result in an over-representation of clustered escape genes. We then tested whether classes of genes were random in their distribution relative to each other and overall confirmed that XCI statuses were not distributed randomly across the X chromosome (Table 1; *P*-value = 0.0083; see Materials and methods). Genes of the same XCI status tended to be located adjacent to each other along the linear chromosome while genes of different XCI statuses were less frequently adjacent to each other than would be expected by chance alone. Variable escape genes tend to have an intermediate level of average genic %Xi expression and while there is not a clear biological boundary that can be used to separate variable escape genes based on average genic %Xi expression, variable escape genes with lower average genic %Xi expression tended to be those adjacent to subject genes on the linear chromosome. Variable escape genes with higher average genic %Xi expressions tended to be those adjacent to escape genes on the linear chromosome (note that locations on the linear chromosome are shown in Figure 2 while rank based on average genic expression are shown in Figure 3A). Overall, the expression pattern of adjacent genes influenced both the likelihood that a gene would be expressed from the Xi and also the expression level, suggesting a substantial contribution of the neighborhood to expression patterns, perhaps reflecting the ability of escape genes to interact with each other [8].

**Table 1:** Chi-square test for neighbor analysis

**Combination of XCI statuses**	**Observed**	**Expected**	**Chi-square statistic = (observed-expected)**^**2 **^**expected**	**Standardized residual = observed-expected expected**^**1/2**^
Escape and escape	39	6	181.5	13.5
Variable escape and variable escape	112	44	105.1	10.3
Subject and subject	258	173	41.8	6.5
Escape and variable escape	19	34	6.6	-2.6
Subject and escape	16	67	38.8	-6.2
Subject and variable escape	53	174	84.1	-9.2

### Population and/or cell line-specific XCI is present across the X chromosome

**Figure 5:**
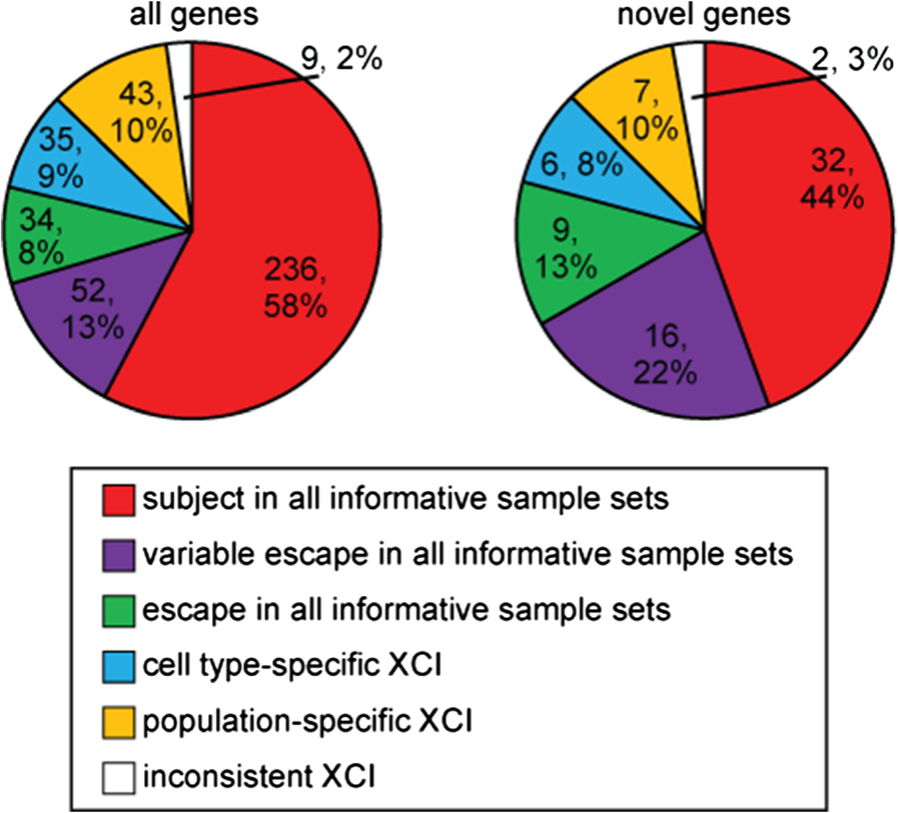
**The majority of genes show the same XCI status in all informative sample sets.** X-linked genes are mostly subject to XCI (red), show variable escape (purple) or escape from XCI (green) in all sample sets. Three classes of genes show a different XCI status in at least one informative sample set: population-specific XCI (orange), cell line-specific XCI (blue) and inconsistent XCI across the informative sample sets (white). At least five females were required to be informative in each informative sample set for a gene to be considered as differing between sample sets. Novel genes are included within the 'all genes’. The decision tree used to classify genes is shown in Additional file 13.

Nearly one-third of genes examined showed variable escape from XCI, although it is unclear why these genes that variably escape XCI are subject in some females yet escape in others. We evaluated the XCI status of each gene in all group 1 and group 2 females and found that no female showed an over-representation of escape amongst the variable escape genes (data not shown). This suggests that there are not individual females who are predisposed to expression from the Xi, in agreement with previous DNA methylation [14] and expression [10] studies that concluded that variable escape from XCI is not the result of overall epigenetic variations between females. Given our previous finding that XCI can differ between tissues [13], we explored the effect of using three sample sets to determine XCI status, and the possibility that differences in XCI might exist between sample sets. We required a sample set to have at least five informative females to ensure robust categorization, and then determined the XCI status in each sample set separately. As expected, the majority of genes (n = 237, 58%) were subject to XCI in all sample sets while 8% (n = 33) escaped from XCI in all informative sample sets. Eleven percent (n = 43) of genes showed a pattern of XCI dependent on the population of the sample set (Figure 5). Differences in XCI between populations, as determined by the ratio of male:female expression level, have previously been detected, with the YRI samples showing more escape from XCI than the CEU samples [25]. In our study, 72% (n = 31) of genes with population-specific XCI in the YRI population showed more escape from XCI than the CEU/FIB population; however, overall the two LCL sample sets (CEU and YRI) had nearly identical proportions of genes that escaped from XCI (CEU, 12%; YRI, 11%) and were subject to XCI (CEU, 65%; YRI, 64%). Comparatively, the FIB sample set had the lowest degree of escape from XCI (8%) and the highest proportion of genes subject to XCI (75%). The higher degree of escape from XCI in LCLs may in part be due to aberrant DNA methylation reported in LCLs [29]. These LCLs have been in culture for extended periods of time and therefore may show escape from XCI at a subset of genes that are usually subject to XCI, potentially analogous to the increased degree of escape from XCI previously observed in somatic cell hybrids [30]. Our previous analysis of XCI status in blood using DNA methylation [13] found blood to be the tissue with the least escape from XCI, suggesting that any reactivation observed in the LCLs in this study is more likely due to culture than tissue of origin. Only 2% of genes (inconsistent XCI, n = 9) showed a pattern of XCI that could not be classified as consistent across tissues or population or cell line-specific XCI and these genes may represent genes with both population- and cell line-specific XCI. Additionally, it should be noted that differences in the transformation processes used to create the CEU and YRI cell lines may contribute to differences in %Xi expression between them [31]. Any discussion of escape from XCI raises the question as to whether escape is truly a resistance to the spread of silencing or reactivation of a gene after it was initially subject to XCI. The escape from XCI associated with population-specific and cell line-specific XCI observed in this study is likely a combination of these two possibilities and further examination of genes that consistently escape from XCI compared to those that differ will be needed to determine how escape from XCI is established.

### AI in females with random XCI fails to reveal X-linked imprinted genes

Studies of females with X-chromosome aneuploidies have described different phenotypic outcomes based on the parent of origin of the X chromosome [32,33]. Recently, a brain-specific X-linked imprinted gene, *MAP7D2*, has been reported [34]. Those females in which there was not enough skewing of XCI to convert AIs into XCI status (group R females) provided an opportunity to consider the presence of X-linked imprinted genes. One sample (WG2121) was classified as a group R female; however, as can be seen in Figure 1A, she has a much higher average AI than the subject training set (0.2148) and was found to be a significant outlier (*P*-value <0.05, Z = 3.273891) from the other group R females and was therefore excluded from further analysis. Group R females show random XCI, meaning that in some cells the maternal X chromosome is the Xi and in others the paternal X chromosome is the Xi. Therefore, regardless of whether a gene is subject to XCI or escapes from XCI, a bi-allelic expression pattern will be observed when the sample is examined as a whole. An exception would be an X-linked imprinted gene that would show mono-allelic expression based on the parent of origin. Individual genes were classified as mono-allelic when the average AI was above the AI at which >99.5% of autosomal probes were found (Additional file 9), and genic mono-allelic expression was calculated by determining the percentage of informative females that showed mono-allelic expression (Figure S4A in Additional file 10). Overall, 462 genes were informative in at least one group R female and 25 genes had one or more females classified as having mono-allelic expression (Figure S4B in Additional file 10). Of the 251 genes that were informative in more than five females, only two (*PPEF1* and *AMOT*) showed variable allelic expression. Studies have found that the X-linked genes *Esx*, *Ftx*, *Jpx*, *Plac1* and *Zcchc13* show imprinting in the mouse [35,36]. None of the females in this study were informative at *ESX1*, and *ZCCHC13* was not present on the array; however, informative females were present at *FTX*, *JPX* and *PLAC1*. All of *FTX*, *JPX* and *PLAC1* were shown to have bi-allelic expression in all informative group R females, suggesting that humans are not imprinted for these genes. The one gene (*MAP7D2*; Figure S4B in Additional file 10) for which there was previous evidence of X-linked imprinting in humans was found to show population-specific XCI (Figure 2; in the group 1 and 2 females) - it escaped from XCI in the CEU sample set but was subject in the YRI sample set. In the group R females, *MAP7D2* showed bi-allelic expression in six females and mono-allelic expression in only one female, resulting in classification as a bi-allelically expressed gene. Furthermore, in this data set, there was no significant difference between male and female *MAP7D2* expression levels (data not shown), supporting that maternal imprinting is not occurring. Therefore, while we cannot address the brain-specific expression status of *MAP7D2* or other X-linked genes, our evidence suggests that X-linked imprinting in the samples analyzed is not common.

## Conclusions

As would be expected for a system that is believed to have evolved to achieve dosage compensation in 46, XX females and 46, XY males, the majority (58%) of genes were found to be subject to XCI in all sample sets. Unexpectedly, we detected a continuum of expression from the Xi along with differences in histone modifications related to the level of Xi expression. Consistent escape from XCI was observed for 8% of genes, but we also observed that many genes escape from XCI in a subset of females and not others. This variable escape from XCI was seen to differ between populations at 43 genes that may reflect differences in XCI caused by sequence-specific differences and/or differences in cell culture. Similarly, the 35 genes with cell line-specific XCI could reflect differences in XCI based on the cellular origin of the cells or might reflect reactivation of X-linked genes in LCLs as a result of the immortalization process and/or extended time in culture. Overall, two-thirds of genes that variably escape from XCI showed a wide range of %Xi expression. This suggests that bimodal variable escape from XCI, in which some females are subject to XCI and others show high levels of escape, is rare and that variable escape from XCI is usually characterized by a continuum of Xi expression levels. The study of how genes escape XCI through the presence of yet unknown *cis*-acting DNA sequences requires that an XCI status be determined for as many genes as possible. Overall, we were able to determine the XCI status of 115 genes where none was previously known. This knowledge is a valuable addition to the ever expanding list of genic XCI statuses and the potential effect these genes have on phenotype differences between males and females and individuals with abnormal numbers of X chromosomes.

## Materials and methods

### Sample preparation and expression array hybridization

The samples and their processing have been previously reported for Caucasian (CEU) samples [1]. Here we also utilized 60 unrelated YRI HapMap samples, of which 56 were successfully grown (all phase 1 and 2 samples except GM18862, GM19116, GM19152, GM19153). The DNA and RNA extraction, cDNA synthesis and parallel analysis for allelic expression at heterozygous sites were carried out on Illumina Human Human1M-Duo (Illumina Inc., San Diego, CA, USA) essentially as previously described [1]. The fibroblast data are from an extension of our previously study [2] including Caucasian parent-offspring fibroblast trios. All LCLs were obtained from Coriell (Camden, NJ, USA) and fibroblast cell lines were also obtained from Coriell and the McGill Cellbank (Montreal, QC, Canada). The allele ratio skewing caused by differences in signal intensities between genomic DNA and cDNA were corrected by applying a polynomial regression model as previously described [37]. The data discussed in this publication have been deposited in NCBI’s Gene Expression Omnibus (GEO) [38] and are accessible through GEO Series accession number GSE26286.

### Probes excluded from XCI status analysis

Two sets of probes were removed from the analysis, those that showed a low total cDNA exThe average expression level (sum of both cDNA channels) and average AI was determined for all uninformative females (CEU, n = 30; YOR, n = 31; FIB, n = 38), then graphed and a one phase decay linear regression performed. The Tau for each sample set was then determined (CEU = 1,020, YRI = 4,174, FIB = 4,331). Only probes with a total cDNA expression greater than the sample set specific Tau (Additional file 4) were used in further analysis. Details of Tau thresholds, including the proportions of probes removed due to a low total cDNA expression level are in Additional files 6 and 11. A total of 978 probes that consistently showed a high genomic DNA ratio (>0.7) in at least 50% of informative females in one sample set were also excluded from analysis in all sample sets, as the anticipated high levels of mono-allelic expression could readily exceed this value and confound the classification of genic XCI status.

### Classification of group 1 females

The average genic AI for each female was only calculated for genes for which at least two probes were informative in that female. The level of skewing of XCI in each female was determined by the average genic AI from those genes from the subject training set (Additional file 1) that were informative in that female. Females with an average genic AI from the subject training set greater than the AI at which only 0.5% of autosomal probes (previously published data [1,2]; Wagner *et al.*, manuscript in preparation) were found (CEU = 0.3587, YRI = 0.3083, FIB = 0.2635) were classified as group 1 females (Figure 1A).

In samples where XCI was not completely skewed, the Xi would be the maternal X chromosome in some cells and the paternal X chromosome in others. To calculate the AI that would corresponded to 10% expression from the Xi but taking into account the mixed population of cells in the group 1 females, the level of skewing of XCI (listed in Additional file 12) needed to be calculated for each female using Equation 1:

$$\mathrm{AI}=\mathrm{ABS}\left|\frac{\left[\left(\mathrm{\%Xa}\exp.\right)\times \left(\%\;\mathrm{of}\;\mathrm{type}\;1\;\mathrm{cells}\right)\right]+\left[\left(\mathrm{\%Xi}\exp.\right)\times \left(\%\;\mathrm{of}\;\mathrm{type}\;2\;\mathrm{cells}\right)\right]}{\left\{\left(\%\;\mathrm{of}\;\mathrm{type}\;1\;\mathrm{cells}\right)\times \left[\left(\mathrm{\%Xi}\exp.\right)+\left(\mathrm{\%Xa}\exp.\right)\right]\right\}+\left\{\left(\%\;\mathrm{of}\;\mathrm{type}\;2\;\mathrm{cells}\right)\times \left[\left(\mathrm{\%Xi}\exp.\right)+\left(\mathrm{\%Xa}\exp.\right)\right]\right\}}-0.5\right|$$

For example, GM11882 from the CEU sample set had an average genic AI from genes in the subject training set of 0.3891, which translates to being 88.91% skewed. Therefore, 88.91% of cells will have one X chromosome as the Xi (type 1 cells) and 11.09% of cells will have the other X chromosome as the Xi (type 2 cells). Assuming that the expression from the Xa = 100% and the expression from the Xi = 10% (the typical expression cutoff for genes subject to XCI), this corresponds to an AI = 0.3184 (see example below).

$$\begin{array}{l} \mathrm{AI}=\mathrm{ABS}\left|\frac{\left[\left(100\%\right)\times \left(88.91\%\right)\right]+\left[\left(10\%\right)\times \left(11.09\%\right)\right]}{\begin{array}{l} \left\{\left(88.91\%\right)\times \left[\left(10\%\right)+\left(100\%\right)\right]\right\}\\ \;+\left\{\left(11.09\%\right)\times \left[\left(10\%\right)+\left(100\%\right)\right]\right\} \end{array}}-0.5\right|\\ \mathrm{AI}=\mathrm{ABS}\left|0.1816-0.5\right|\\ \mathrm{AI}=0.3184 \end{array}$$

### Conversion of AI into XCI status for group 1

Genes with an average AI less than that corresponding to 10% Xi expression were classified as being subject to XCI in that female. While this threshold varied between females based on skewing of XCI, the thresholds used to divide genes into three levels of escape from XCI were the same across all group 1 females from a given sample set (listed in Additional file 9). The AI below which 90% of autosomal probes were found was used to define the highest level of escape from XCI (E_1_) for the group 1 females. The AI that 95% of autosomal probes were below was then used to define the middle level of escape from XCI (E_2_) for the group 1 females. The thresholds listed in Additional file 9 were then used to predict an XCI status for each gene in each informative group 1 female. In each sample set, the percentage of group 1 females that escaped from XCI was calculated. Using the cutoffs first established by [10], genes in which 78 to 100% of informative females escaped from XCI were classified as escaping from XCI, genes in which 0 to 22% of informative females escaped from XCI were classified as being subject to XCI, and genes in between were defined as variably escaping from XCI.

### Division of group 2 females from group R females

As with the group 1 females, it was necessary to adjust for skewing of XCI in the non-group 1 females. However, we had an expectation that in females with completely random XCI (skewing = 50%) we would not be able to translate AI into an XCI status. In such an individual, all genes, regardless of if they were subject to or escaping from XCI, would be expressed from both alleles. To determine which females were skewed enough to differentiate between genes escaping from XCI (and therefore expressed from both X chromosomes) and genes subject to XCI (expressed from only the Xa), we performed a linear regression between each non-group 1 female and the average genic AI from the group 1 females in that sample set. Only genes that showed a consistent pattern of XCI (subject to or escaping from XCI) in group 1 females were used so as to provide the best subset of genes for determining skew. Additional file 5 lists all non-group 1 females and classifies those with a significant (*P*-value ≤0.05) linear regression correlation into group 2 females while those that are not significantly correlated are classified as group R females.

### XCI thresholds in group 2 females

In group 1 females, the E_1_:E_2_ and E_2_:E_3_ boundaries were the same in all females regardless of skewing, whereas the E_3_:S boundary of 10% Xi expression changed between females. However, due to the highly variable level of skewing of XCI in the group 2 females, the E_1_:E_2_ and E_2_:E_3_ boundaries in addition to the E_3_:S boundary were adjusted for skewing. In group 2 females, the formula of the linear regression line was used to convert the E_1_:E_2_ and E_2_:E_3_ boundaries from the group 1 level to a female-specific group 2 level. The AI that corresponded to 10% Xi expression, taking into account skewing of XCI, was also adjusted using the formula of the linear regression line. The dotted lines in Figure S1A,B in Additional file 2 illustrate examples of the E_1_:E_2_ and E_2_:E_3_ boundaries, and the shading represents the range of AIs used to predict XCI in that group 2 female. A complete list of all boundaries can be found in Additional file 5.

### Conversion of AI into Xi expression as a ratio of Xa expression

The same formula (formula 1) used to calculate the level of skewing in group 1 females was used to translate AIs into %Xi expression simply by solving for %Xi expression. In doing so it was necessary to assume that the level of expression for the Xi would be the same regardless of which (maternal or paternal) X chromosome was the Xi. When a %Xi was predicted to be below zero, likely due to an underestimation of skewing, it was assigned a %Xi equal to zero.

### Chi-square analysis of distribution of XCI statuses along the X chromosome

In order to determine if the distribution of XCI statuses was random a Chi-square analysis was performed excluding the PAR1 region and the number of observed versus expected combinations of XCI statuses determined. Significance was determined at a *P*-value of 0.05.

### Analysis of epigenetic features

Histone ChIP AI was performed as follows. LCLs were grown to log phase (10^6^ cells/ml maximum density) in 40 ml of media then cross-linked with 1% formaldehyde at room temperature for 10 minutes. After quenching with glycine for 5 minutes (125 mM glycine per ml of media), the cells were washed twice with ice-cold phosphate-buffered saline. Cells were collected after each wash by centrifugation at 2,000 *g* for 5 minutes. Cell pellets were flash frozen and stored at -80°C. Frozen pellets were thawed and cells were lysed in Farnham lysis buffer (5 mM PIPES pH 8.0, 85 mM KCl, 0.5% NP-40 and protease inhibitors) for 10 minutes on ice. After centrifugation and wash with 1 ml of RIPA buffer containing 50 mM Tris HCl pH 8, 150 mM NaCl, 1% NP-40, 0.5% sodium deoxycholate, 0.1% SDS and protease inhibitors, lysates were then diluted with 500 μl of RIPA buffer. Cells were sonicated in non-stick tubes under conditions optimized to yield soluble chromatin fragments in a size range of 100 to 250 base pairs. Chromatin from 40 million cells was sonicated for 10 minutes using a Branson 250 sonicator at 20% power amplitude (pulses of 10 s on and 30 s off). Lysate was cleared by centrifuging at 12,000 *g* for 10 minutes at 4°C to eliminate cellular debris. Chromatin was then flash frozen and stored at -80°C or used immediately for the next step. Before each immunoprecipitation, chromatin was pre-cleared with 50 μl of prewashed ProteinA-magnetic beads (Invitrogen, Carlsbad, CA, USA; 100-02D) to avoid non-specific binding. Immunoprecipitation was carried out for 12 h by rotation at 4°C in 500 μl of chromatin/RIPA buffer supplemented with protease inhibitor cocktails (Roche Diagnostics, Indianapolis, IN, USA ; 04 693 159 001) and PMSF. We used 10 to 100 million cells and 2 to 20 μg of the following antibodies for each assay: H3K4me1 (Diagenode , Denville, NJ, USA ; #pAb-037-050), H3K4me3 (Diagenode; #pAb-003-050), H3K27ac (Abcam , Cambridge, ENG, UK; #ab4729), H3K27me3 (Millipore, Darmstadt, Germany); #07-449), H3K36me3 (Abcam, #ab9050). After overnight incubation, samples were rotated with 100 μl of prewashed ProteinA-magnetic beads at 4°C for 1 h. The beads were then collected by brief centrifugation at 2,000*g* following by use of a magnetic rack. Beads were washed five times with 1 ml of LiCl wash buffer (100 mM Tris pH7.5, 500 mM LiCl, 1% NP-40, 1% sodium deoxycholate) by resuspending the beads and keeping on ice for 10 minutes. Bound chromatin was eluted from the beads using 200 μl of elution buffer (50 mM Tris-HCl, pH 8.0, 10 mM EDTA, 1.0% SDS) by incubation at 65°C for 1 h with vortexing every 15 minutes followed by centrifugation at 14,000 *g* at room temperature for 3 minutes. The eluted chromatin and the input sample were incubated at 65°C overnight after adding 0.2 M of NaCl to remove crosslinks. Samples were then treated with RNase at 37°C for 30 minutes and digested with proteinase K at 55°C for 1 h. Immunoprecipitated DNA was purified using QIAquick PCR Purification Kit (QIAGEN, Toronto, ON, Canada; 28104) and eluted in 30 μl. Enrichments of known ChIP-seq peaks were validated using real-time PCR experiments for each antibody. Primers were designed to genomic sites known to bind H3K4me1, H3K4me3, H3K27ac, H3K27me3, H3K36me3 or none of them. Samples that showed expected enrichment were treated like double-stranded cDNA samples and assessed for allelic imbalance on Illumina BeadChips. The data discussed in this publication have been deposited in NCBI’s GEO [38] and are accessible through GEO Series accession number (GSE51272).

The LCL panel we used for this analysis consisted of five female LCL samples: GM12873, GM12892, GM18502, GM18508, and GM19240. Each sample was assessed via the aforementioned histone ChIP AI protocol for H3K4me1 and H3K4me3 AI using 1M, 2M and 2S Illumina BeadChip genotyping arrays. GM19240 was further assessed for H3K27ac, H3K27me3 and H3K36me3 AI using 2M and 2S Illumina BeadChip genotyping arrays. AI values for heterozygous SNPs were calculated as previously described for cDNA analyses. Absolute AI values for heterozygous SNPs lying within 1 kb of TSS sites, and across transcripts, of genes in each of the four XCI gene classes described were used to generate histograms of average AI for each histone modification and total chromatin AI (five histone modifications combined). Significance of differences in mean AI between gene classes was assessed via two-tailed *t*-tests, and corrected for multiple testing.

### Determination of Y chromosome and autosomal homology

All genes predicted to escape from XCI were submitted to the Nucleotide Basic Local Alignment Search Tool and compared against the entire genome (all assemblies scaffolds) [26]. Those with an identity score ≥80% to either the Y chromosome or the autosomes were classified as having homology to Y chromosome and/or the autosomes.

### Classification of population and cell line-specific XCI

The number of informative females in each sample set was determined and those with fewer than five informative females excluded. A decision tree (outlined in Additional file 13) was then used to classify genes as either having an XCI status that was consistent across all informative sample sets or as differing between sample sets.

### XCI status in group R females

A Grubb’s outlier test (significance at *P*-value <0.05) based on the average genic AI was performed in each sample set for all group R females. Only WG2121 from the FIB sample set was found to be a significant outlier and was therefore excluded from further analysis. For group R females the sample set specific AI at which more than 99.5% of autosomal probes were found (Additional file 9) was used to define mono-allelic expression. Genes with an AI above this threshold were classified as showing mono-allelic expression, below as bi-allelic expression. YRI and FIB sample sets were scaled as with the group 1 and group 2 females.

## Abbreviations

AI: Allelic imbalance; CEPH: Centre d’Etude du Polymorphisme Humain; CEU: CEPH HapMap population sample set; ChIP: Chromatin immunoprecipitation; FIB: Primary fibroblast sample set; GEO: Gene Expression Omnibus; Histone ChIP AI: Histone chromatin immunoprecipitation allelic imbalance; LCL: Lymphoblast cell line; PAR1: Pseudoautosomal region 1; SNP: Single nucleotide polymorphism; TSS: Transcription start site; Xa: Active X chromosome; XCI: X-chromosome inactivation; Xi: Inactive X chromosome; YRI: Yoruban HapMap population sample set.

## Competing interests

The authors declare that they have no competing interests.

## Authors’ contributions

BG carried out the array experiments and analysis of autosomal probes. AMC performed the X chromosome data analysis and drafted the manuscript. TP and CJB conceived of the study. NL and VA carried out the histone ChIP AI analysis. All authors read and approved the final manuscript.

## Supplementary Material

Additional file 1: Table S1 Subject training set genes. List of 177 genes in the subject training set. All genes were previously found to be subject to XCI by expression analysis in somatic cell hybrids [10] (expression in 0 to 22% of examined somatic cell hybrids) and were also subject to XCI based on DNA methylation analysis in all tissues [13]. The expression of a subset of genes, marked with an asterisk, as examined in fibroblasts [10] also supports that these genes are subject to XCI (average of less than 10% Xi expression). Click here for file

Additional file 2: Figure S1 Identification of females with skewed XCI. Conversion of AI into XCI status in a group 2 female with a high degree of skewing **(A)** compared to a female with low skewing **(B)**. The line demonstrates the linear regression between the female analyzed and the average AI from the CEU group 1 females (subject and escape genes only). The horizontal shading denotes the ranges of AI that correspond to the XCI statuses in the group 2 female: dark green (E_1_), bright green (E_2_), light green (E_3_) or red (subject to XCI). The lower degree of skewing of XCI **(B)** results in a condensed range of escape from XCI. For all group 2 females, the boundary between E_3_ and S was determined using the AI at which there was 10% expression from the Xi once corrected for skewing. A complete list of boundaries can be found in Additional file 5. **(C)** The linear regression between the average group 1 female AI and group R females was not significant and therefore AIs in group R females could not be converted in XCI statuses. A complete list of group R females can be found in Additional file 5. Click here for file

Additional file 3: Table S2 Escape training set genes. List of 43 genes in the escape training set. All genes were previously found to be escape from XCI by expression analysis in somatic cell hybrids [10] (expression in 78 to 100% of examined somatic cell hybrids) and also escaped from XCI based on DNA methylation analysis in all tissues [13]. The expression of a subset of genes, marked with an asterisk, as examined in fibroblasts [10] also supports that these genes escape from XCI (average of more than 10% Xi expression). Those genes located in the PAR1 are marked with a pound sign. Click here for file

Additional file 4: Figure S2 Minimum cDNA probe intensity thresholds differ in each sample set. **(A,B)** CEU sample set, **(C,D)** YRI sample set, **(E,F)** FIB sample set. **(A,C,E)** The average expression (both cDNA channels) and AI were determined for all uninformative females (CEU, n = 30; YRI, n = 31; FIB, n = 38), then graphed and a one phase decay linear regression performed. The Tau for each population was then determined (solid black line in **(B,D,F)**) and the 95% confidence interval also plotted (dotted black line in **(B,D,F)**). Only probes with a total cDNA expression greater than Tau were used in further analysis. Details of Tau thresholds are in Additional files 6 and 11. Click here for file

Additional file 5: Table S3 Thresholds of AI for conversion into XCI status following linear regression in non-group 1 females. Group 2 females show a significant linear regression with the average AI from the group 1 females whereas group R females are not significant and therefore AIs cannot be converted into XCI status using the slope of the linear regression line. Click here for file

Additional file 6: Table S4 The majority of probes are removed due to a low cDNA probe intensity. Click here for file

Additional file 9: Table S6 AI thresholds used to translate XCI status for group 1 females from each sample set. Within each sample set the E_1_:E_2_ and E_2_:E_3_ boundaries are the same for all individuals while the E_3_:S boundary differs based on the degree of skewing in each female. The AI that corresponded to 10% Xi expression was calculated based on the degree of skewing in each female. The AI for each group 1 female can be found in Additional file 12. Click here for file

Additional file 12: Table S8 AI at 10% Xi expression for each group 1 female. Click here for file

Additional file 7: Table S5 List of all genic XCI statuses determined. The gene name, if the gene is a member of the escape or subject training set, the total number of informative females, the percentage of which escape from and the genic XCI status are listed along with the average %Xi and the standard deviation of the %Xi for each gene. Genic XCI status cells are colored, subject to XCI (red), variable escape from XCI (purple) and escape from XCI (green). Genes are listed from Xp to Xq. Click here for file

Additional file 8: Figure S3 Histone ChIP AI of combined histone modifications is highest at genes subject to XCI. Error bars represent the standard error of the mean while the color indicates the assigned genic XCI status. Genes that escape XCI and have a %Xi expression within the PAR1 range (dark green), genes that escape XCI and have a %Xi expression outside the PAR1 range (green), genes that are subject to XCI and have a %Xi expression greater than 5% Xi (red) and genes that are subject to XCI and have a %Xi expression less than 5% Xi (dark red). Significant differences between means are shown as asterisks (*P-value 0.05 to 1.0 e-5, **P-value 1.0 e-5 to 1.0 e-15, ***P-value <1.0 e-15). All *P*-values were corrected for multiple comparisons. Click here for file

Additional file 13: Figure S5 Decision tree to determine XCI status across sample sets. In order to compare XCI status between the three sample sets a standard set of yes/no questions was devised. To begin (black rectangle in the center) the two LCL sample sets (CEU and YRI) are examined, then the FIB sample set is brought in to determine if differences in XCI status were the result of population or cell line differences. In total, six different cross-sample set XCI statuses were defined: subject in all sample sets (red), VE (variable escape) in all sample sets (purple), escape in all sample sets (green), population-specific XCI (orange), cell line-specific XCI (blue), population and cell line-specific XCI (blue and orange stripes) and inconsistent XCI between samples sets (white). Click here for file

Additional file 10: Figure S4 Allelic expression analysis in group R females reveals no evidence for X-linked imprinting. **(A)** Allelic expression bias in individual informative females with each column representing one gene (only genes with at least one informative female are included) and each row a single female (group R females only) from the three sample sets. The allelic expression bias of each female is either mono-allelic (red) or bi-allelic (green). The genic expression status was determined by calculating the percentage of females that were bi-allelic for each gene: 0 to 22% of informative females bi-allelic, genic bias is mono-allelic (red); 22 to 78% of informative females bi-allelic, genic bias is variable allelic (purple); or 78 to 100% of informative females bi-allelic, genic bias is bi-allelic (green). **(B)** Distribution of AIs observed for every gene with at least one female with mono-allelic expression. For each gene, informative females are represented with a different shape based on the sample set (CEU, square; YRI, circle; FIB, triangle) and color based on allelic expression status (red, mono-allelic; green, bi-allelic). Below the genic allelic-expression status is given (bi = bi-allelic, VA = variable allelic, mono = mono-allelic). *MAP7D2*, the previously reported X-linked gene, is shown to the far right. Click here for file

Additional file 11: Table S7 Minimum probe intensities are comparable between males and females. Tau was also determined for all uninformative probes in males for each sample set. Click here for file
