# Supplementary figures and images for: Analysis of expressed SNPs identifies variable extents of expression from the human inactive X chromosome
Source: Genome Biol. 2013 Nov 1;14(11):R122. doi: 10.1186/gb-2013-14-11-r122 (PMC4053723; doi:10.1186/gb-2013-14-11-r122)

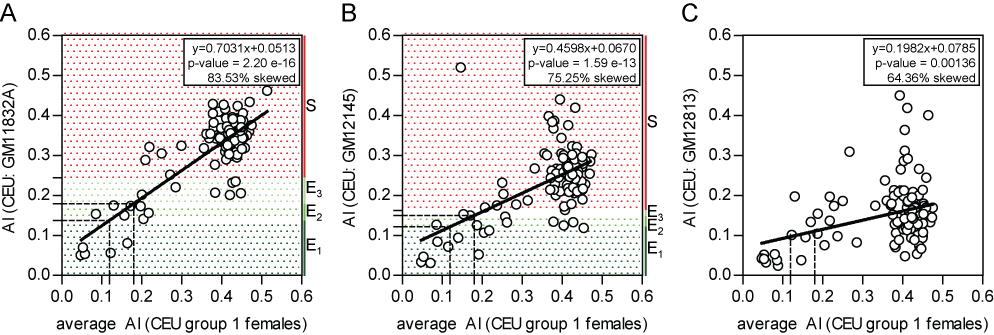

Supplement: Additional file 2: Figure S1 — Identification of females with skewed XCI. Conversion of AI into XCI status in a group 2 female with a high degree of skewing (A) compared to a female with low skewing (B). The line demonstrates the linear regression between the female analyzed and the average AI from the CEU group 1 females (subject and escape genes only). The horizontal shading denotes the ranges of AI that correspond to the XCI statuses in the group 2 female: dark green (E1), bright green (E2), light green (E3) or red (subject to XCI). The lower degree of skewing of XCI (B) results in a condensed range of escape from XCI. For all group 2 females, the boundary between E3 and S was determined using the AI at which there was 10% expression from the Xi once corrected for skewing. A complete list of boundaries can be found in Additional file 5. (C) The linear regression between the average group 1 female AI and group R females was not significant and therefore AIs in group R females could not be converted in XCI statuses. A complete list of group R females can be found in Additional file 5. [file gb-2013-14-11-r122-S2.png]

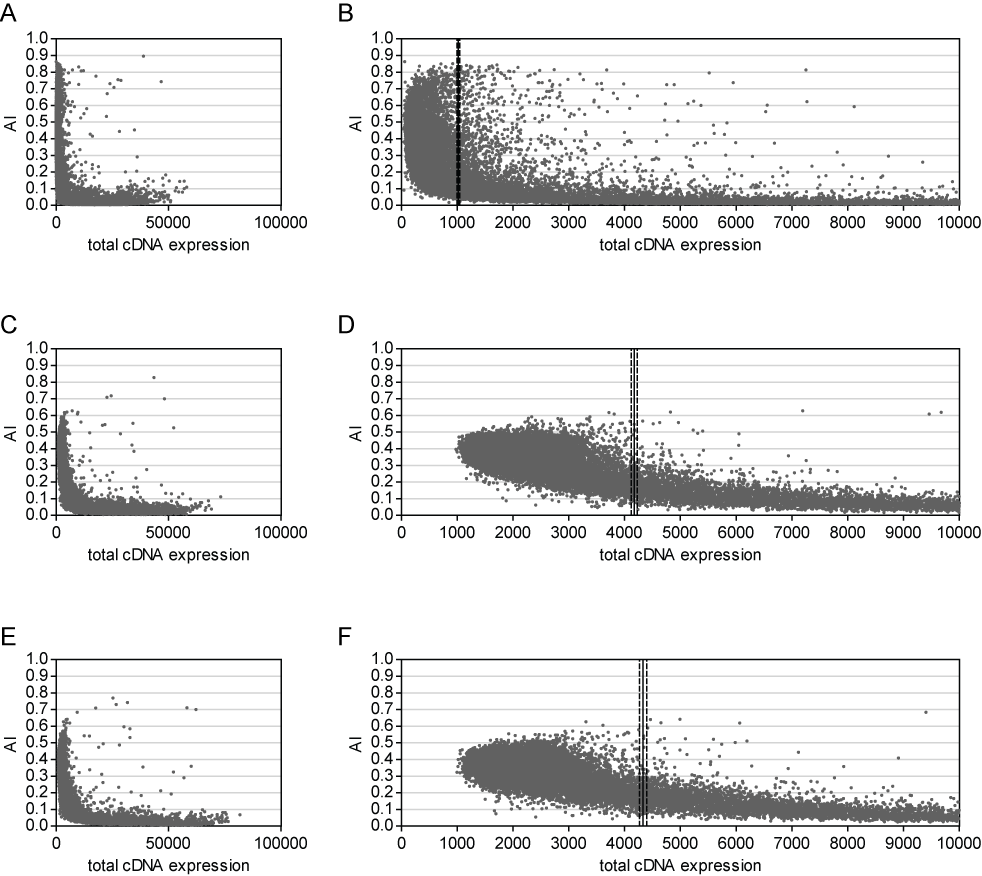

Supplement: Additional file 4: Figure S2 — Minimum cDNA probe intensity thresholds differ in each sample set. (A,B) CEU sample set, (C,D) YRI sample set, (E,F) FIB sample set. (A,C,E) The average expression (both cDNA channels) and AI were determined for all uninformative females (CEU, n = 30; YRI, n = 31; FIB, n = 38), then graphed and a one phase decay linear regression performed. The Tau for each population was then determined (solid black line in (B,D,F)) and the 95% confidence interval also plotted (dotted black line in (B,D,F)). Only probes with a total cDNA expression greater than Tau were used in further analysis. Details of Tau thresholds are in Additional files 6 and 11. [file gb-2013-14-11-r122-S4.png]

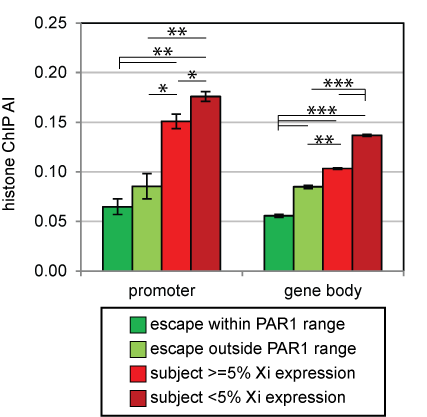

Supplement: Additional file 8: Figure S3 — Histone ChIP AI of combined histone modifications is highest at genes subject to XCI. Error bars represent the standard error of the mean while the color indicates the assigned genic XCI status. Genes that escape XCI and have a %Xi expression within the PAR1 range (dark green), genes that escape XCI and have a %Xi expression outside the PAR1 range (green), genes that are subject to XCI and have a %Xi expression greater than 5% Xi (red) and genes that are subject to XCI and have a %Xi expression less than 5% Xi (dark red). Significant differences between means are shown as asterisks (*P-value 0.05 to 1.0 e-5, **P-value 1.0 e-5 to 1.0 e-15, ***P-value <1.0 e-15). All P-values were corrected for multiple comparisons. [file gb-2013-14-11-r122-S8.png]

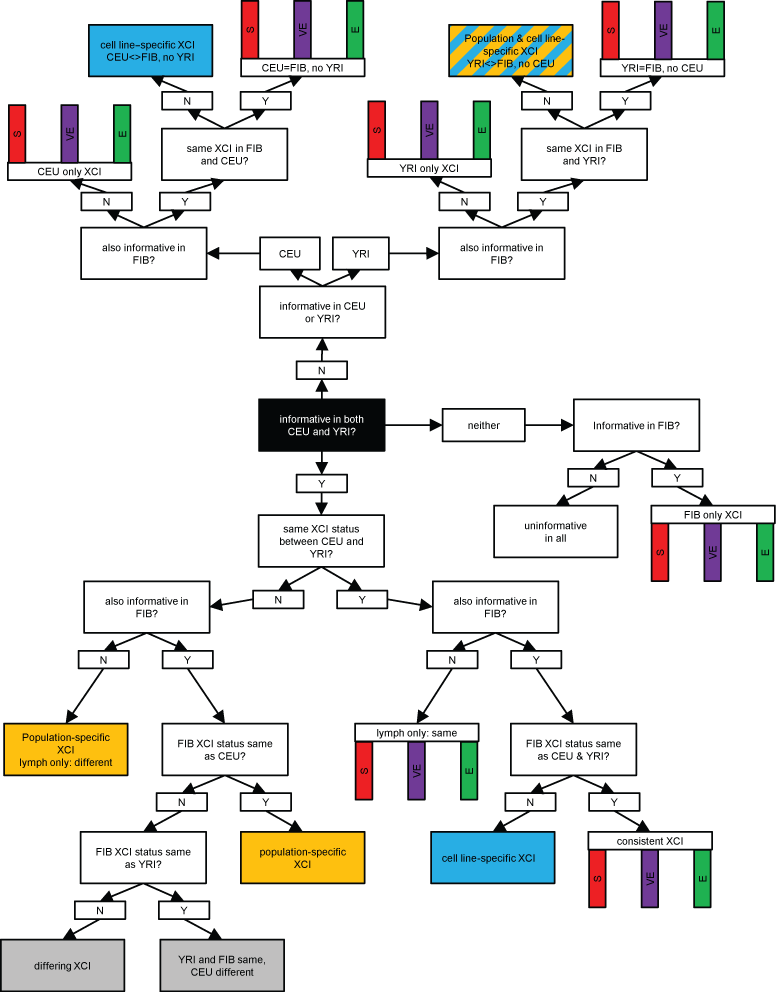

Supplement: Additional file 13: Figure S5 — Decision tree to determine XCI status across sample sets. In order to compare XCI status between the three sample sets a standard set of yes/no questions was devised. To begin (black rectangle in the center) the two LCL sample sets (CEU and YRI) are examined, then the FIB sample set is brought in to determine if differences in XCI status were the result of population or cell line differences. In total, six different cross-sample set XCI statuses were defined: subject in all sample sets (red), VE (variable escape) in all sample sets (purple), escape in all sample sets (green), population-specific XCI (orange), cell line-specific XCI (blue), population and cell line-specific XCI (blue and orange stripes) and inconsistent XCI between samples sets (white). [file gb-2013-14-11-r122-S13.png]

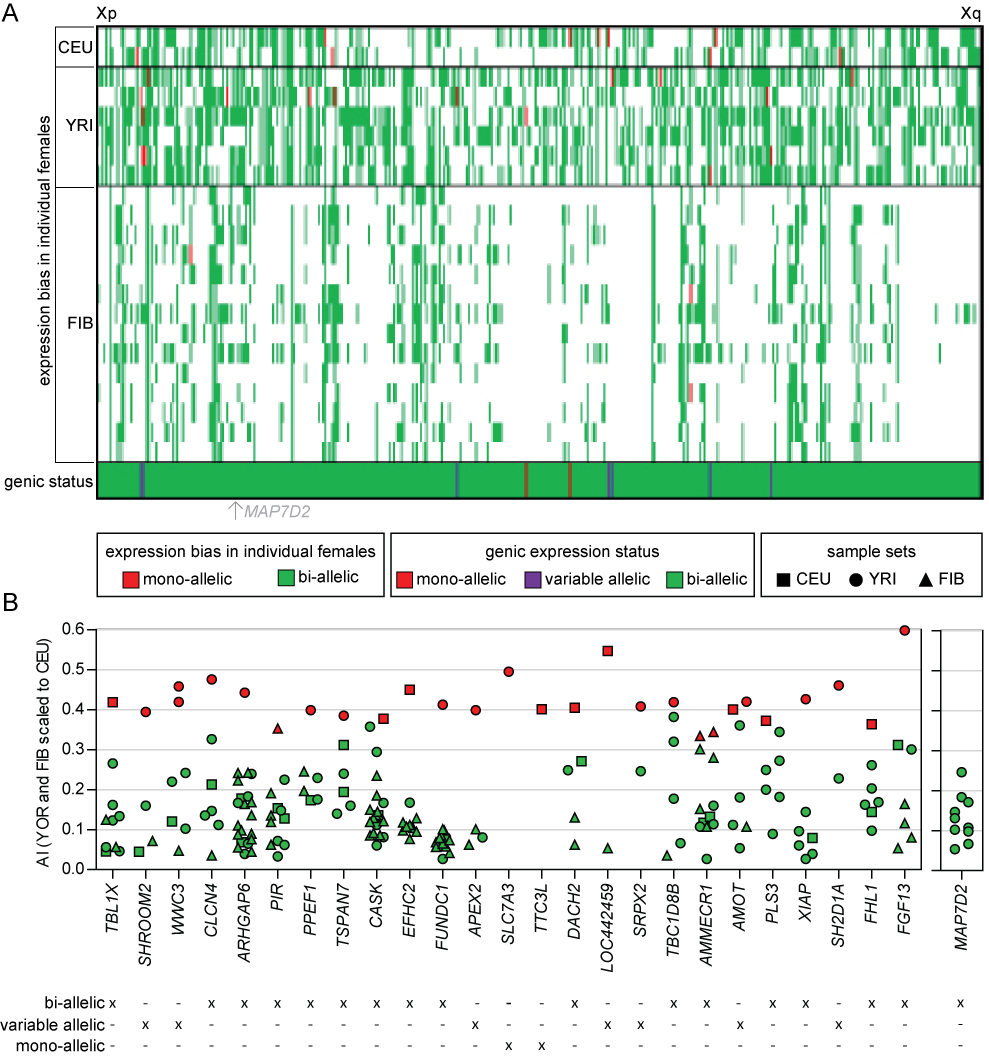

Supplement: Additional file 10: Figure S4 — Allelic expression analysis in group R females reveals no evidence for X-linked imprinting. (A) Allelic expression bias in individual informative females with each column representing one gene (only genes with at least one informative female are included) and each row a single female (group R females only) from the three sample sets. The allelic expression bias of each female is either mono-allelic (red) or bi-allelic (green). The genic expression status was determined by calculating the percentage of females that were bi-allelic for each gene: 0 to 22% of informative females bi-allelic, genic bias is mono-allelic (red); 22 to 78% of informative females bi-allelic, genic bias is variable allelic (purple); or 78 to 100% of informative females bi-allelic, genic bias is bi-allelic (green). (B) Distribution of AIs observed for every gene with at least one female with mono-allelic expression. For each gene, informative females are represented with a different shape based on the sample set (CEU, square; YRI, circle; FIB, triangle) and color based on allelic expression status (red, mono-allelic; green, bi-allelic). Below the genic allelic-expression status is given (bi = bi-allelic, VA = variable allelic, mono = mono-allelic). MAP7D2, the previously reported X-linked gene, is shown to the far right. [file gb-2013-14-11-r122-S10.png]
